# Targeting Calcium Homeostasis in Myocardial Ischemia/Reperfusion Injury: An Overview of Regulatory Mechanisms and Therapeutic Reagents

**DOI:** 10.3389/fphar.2020.00872

**Published:** 2020-06-09

**Authors:** Ruiying Wang, Min Wang, Shuaibing He, Guibo Sun, Xiaobo Sun

**Affiliations:** ^1^Institute of Medicinal Plant Development, Chinese Academy of Medical Sciences & Peking Union Medical College, Beijing, China; ^2^Beijing Key Laboratory of Innovative Drug Discovery of Traditional Chinese Medicine (Natural Medicine) and Translational Medicine, Institute of Medicinal Plant Development, Peking Union Medical College & Chinese Academy of Medical Sciences, Beijing, China; ^3^Key Laboratory of Bioactive Substances and Resources Utilization of Chinese Herbal Medicine, Ministry of Education, Institute of Medicinal Plant Development, Chinese Academy of Medical Sciences & Peking Union Medical College, Beijing, China; ^4^Key Laboratory of Efficacy Evaluation of Chinese Medicine Against Glycolipid Metabolic Disorders, State Administration of Traditional Chinese Medicine, Institute of Medicinal Plant Development, Peking Union Medical College & Chinese Academy of Medical Sciences, Beijing, China; ^5^Key Laboratory of New Drug Discovery Based on Classic Chinese Medicine Prescription, Chinese Academy of Medical Sciences, Beijing, China

**Keywords:** calcium homeostasis, myocardial ischemia/reperfusion injury, calcium signaling pathway, therapeutic reagents, Therapeutic Target Database

## Abstract

Calcium homeostasis plays an essential role in maintaining excitation–contraction coupling (ECC) in cardiomyocytes, including calcium release, recapture, and storage. Disruption of calcium homeostasis may affect heart function, leading to the development of various heart diseases. Myocardial ischemia/reperfusion (MI/R) injury may occur after revascularization, which is a treatment used in coronary heart disease. MI/R injury is a complex pathological process, and the main cause of increased mortality and disability after treatment of coronary heart disease. However, current methods and drugs for treating MI/R injury are very scarce, not ideal, and have limitations. Studies have shown that MI/R injury can cause calcium overload that can further aggravate MI/R injury. Therefore, we reviewed the effects of critical calcium pathway regulators on MI/R injury and drew an intuitive diagram of the calcium homeostasis pathway. We also summarized and analyzed calcium pathway-related or MI/R drugs under research or marketing by searching Therapeutic Target and PubMed Databases. The data analysis showed that six drugs and corresponding targets are used to treat MI/R injury and involved in calcium signaling pathways. We emphasize the relevance of further detailed investigation of MI/R injury and calcium homeostasis and the therapeutic role of calcium homeostasis in MI/R injury, which bridges basic research and clinical applications of MI/R injury.

## Introduction

Myocardial ischemia/reperfusion (MI/R) is an inevitable process in the treatment of cardiovascular diseases such as acute myocardial infarction, thrombolysis, coronary angioplasty, and cardiac arrest (Zhu et al., 2017). MI/R injury refers to a more serious damage caused by ischemic myocardial tissues after blood perfusion is restored. MI/R can aggravate reversible damage to cardiac tissue; it may also promote reversible damage into irreversible damage of cardiac tissue, eventually worsening the patient's condition and even causing death (Fiolet and Baartscheer, 2000). It can be clinically manifested as arrhythmia, decreased cardiac function, and enlarged myocardial necrotic area (Murphy and Steenbergen, 2008). Recently, research has mainly focused on various theories such as calcium overload, energy metabolism disorders, free radical effects, oxidative stress, inflammatory response, and mitochondrial dysfunction (Thind et al., 2015). Although vast studies have reported on the mechanism underlying MI/R injury, the clinical transformation is limited and the therapeutic effects are not ideal. At present, there are few clinical treatments for MI/R injury, mainly through ischemic post-conditioning and drugs of anti-free radicals, dilating blood vessels, and reducing calcium overload. Therefore, further exploring the mechanism of MI/R injury and developing more effective anti-MI/R drugs are necessary (Turer and Hill, 2010; Ibanez et al., 2015). Calcium homeostasis is particularly important for myocardial cell structure and function. So, it is of significance to focus on some cardiac protective reagents related to calcium overload to treat MI/R injury (Kleinbongard et al., 2012).

Ca^2+^ is an important cytoplasmic signaling molecule in most cellular responses and is mainly distributed in extracellular and intracellular organelles, such as sarcoplasmic reticulum (SR) and mitochondria. Calcium plays a vital role in excitation–contraction coupling (ECC) of heart tissue. When cardiac action potential occurs, extracellular Ca^2+^ enters the cell through L-type Ca^2+^ channels (LTCC), and intracellular Ca^2+^ activates ryanodine receptor 2 (RyR2) and more Ca^2+^ is released from the sarcoplasmic reticulum releases (Marks, 2013). When the intracellular Ca^2+^ is present at a certain level, it binds to the myofilament protein troponin C causing myocardial contraction. On the one hand, sarco (endo) plasmic reticulum Ca^2+^-ATPase (SERCA) recaptures intracellular Ca^2+^ back to SR. On the other hand, sodium/calcium exchanger (NCX) expels Ca^2+^ from the cells and the dissociation of Ca^2+^ from myofilament protein relaxes the myocardial cells (Hadri and Hajjar, 2011). The ECC in myocardial cells can also be attributed to multiple organelles together to maintain intracellular calcium homeostasis. Calcium levels in myocardial cells are regulated by LTCC, NCX, SERCA, RyR2, and mitochondria, which participate in calcium overload during MI/R injury (Landstrom et al., 2017). The study of cardiac calcium homeostasis has always been a hot topic. Common experimental subjects include human induced pluripotent stem cells (hiPSCs), isolated hearts, primary cardiomyocytes and whole animals(Pall et al., 2003; Lee et al., 2011). Experimental methods of calcium homeostasis are also constantly evolving usually through electrophysiological methods, such as field potential and patch clamp; the direct capture technology of intracellular calcium sparks, calcium leakage, and calcium transients; traditional gene and protein qualitative or quantitative techniques for in-depth study (Huffaker et al., 2004; Eden et al., 2016). Continuously updated research methods are conducive to clarifying the role of calcium homeostasis in MI/R injury, thus accelerating drug development for the treatment of MI/R injury to alleviate limitations of clinical drugs.

Calcium homeostasis disorder is regarded as an important treatment target for clinical intervention of MI/R injury. However, there is currently no article that summarizes and introduces the interaction between calcium homeostasis and MI/R injury. Therefore, we review the role of calcium homeostasis in MI/R injury and the contribution of calcium signal pathway-related drugs currently under research or in the market to MI/R injury. Our work aimed to provide strong evidence for further development of MI/R candidate drugs treatment for MI/R injury.

## Calcium Homeostasis

### Calcium Release

During cardiac action potential, Ca^2+^ enters through LTCC, which triggers Ca^2+^ release from SR through RyR2. And FKBP12.6 stabilizes RyR2 and prevents abnormal contraction of cardiomyocytes (Bers, 2008).

#### LTCC

Voltage-gated calcium channels include Cav1 (LTCC), and Cav2 and Cav3, which are non-LTCCs. Cav1.2 and Cav1.3 are co-expressed in multiple tissues (sinus node, atrium, and neurons) and play similar regulatory roles. Cav1.1 and Cav1.4 are expressed only in skeletal muscle and retina, respectively (Striessnig et al., 2010). LTCC channel consists of α1, β, α2δ, γ subunits, and calmodulin. The α1 subunit is the main pore-forming structure with the most significant effect on the LTCC current intensity. As a dual regulator, calmodulin is capable of Ca^2+^-dependent inactivation and facilitation. The T-tubule on the plasma membrane of ventricular myocytes is a typical recessed structure with a large amount of LTCC distribution (Shaw and Colecraft, 2013). At myocardial cell action potential, LTCC is activated by depolarization and opened for extracellular Ca^2+^ into the cell. This enriched T-tubule spatial advantage is helpful for RyR2 of nearby SR to complete calcium-induced calcium release. β2 adrenergic receptor (β2AR) interacts directly with LTCC (Cav1.2); LTCC is regulated by G protein, adenylate cyclase, PKA, PP1, and PP2A, which affects channel activity. Some studies have shown that loss or deficiency of LTCC function can lead to short QT intervals and sudden cardiac death (Shaw and Colecraft, 2013). Cav1.2 subtype blockers, such as nifedipine, verapamil, diltiazem, are clinically called calcium channel blockers and are used for the treatment of hypertension, angina pectoris, and arrhythmia (Kleinbongard et al., 2012). Therefore, LTCC plays a pivotal role in many cardiovascular diseases, and more research is needed to elaborate its specific mechanisms.

#### RyR2

RyR is a homotetrameric structure located on the SR membrane. There are three RyR subtypes distributed in mammals: RyR1 is mainly distributed in skeletal muscle, RyR2 primarily in cardiac muscle tissue (Laver, 2018), and RyR3 mainly in brain tissue. This article mainly introduces RyR2 function of in myocardial tissue. RyR2 is a calcium channel that releases calcium from SR to the cytoplasm after LTCC activation (Dulhunty et al., 2012). The amount of calcium released by SR during a cardiac cycle is related to LTCC current, RyR2 Ca^2+^ sensitivity, and SR Ca^2+^ content (Cully et al., 2018). RyR2 and inositol triphosphate receptor (IP3R) have the same conservative structure, and therefore, may have evolved from a common precursor structure; the common allosteric activation mechanism of both is N-terminus displacement of two adjacent domains (Dulhunty et al., 2012). PKA hyperphosphorylated RyR2 increases calcium-dependent calcium release, leading to diastolic calcium leakage in cardiomyocytes and decreased myocardial contractility. Importantly, FKBP12.6 can stabilize RyR2, thereby preventing excessive calcium release by RyR2 and intracellular calcium homeostasis imbalance (Oda et al., 2015). The role of RyR2 in M/R remains debatable; however, reducing RyR2 activity has a protective effect on cardiomyocytes. Specifically, reducing RyR2 opening before ischemia can reduce the calcium overload (Yang et al., 2015). Studies have shown that during MI/R, RyR2 structure gets damaged, which also accounts for large amount of calcium leaks in SR and calcium overload. Therefore, reducing calcium leakage may be a novel strategy for treating MI/R (Zucchi et al., 1994; Fauconnier et al., 2013).

#### FKBP12.6

The FK506 binding proteins (FKBP) are expressed in cardiomyocytes and act as essential RyR2 regulators. FKBP12 and FKBP12.6 subtypes correspond to low and high RyR2 affinity, respectively. Reportedly, FKBP12.6 and not FKBP12 can inhibit RyR2 activity. FKBP12.6 and RyR2 association keeps RyR2 closed, preventing calcium release from the SR, thereby, ensuring heart relaxation (Xiao et al., 2018). PKA, Ca^2+^-calmodulin–dependent protein kinase II (CaMKII), and phosphatases are involved in RyR2 phosphorylation. PKA-induced RyR2 phosphorylation (Ser-2808) promotes FKBP12.6 and RyR2 dissociation, thereby opening RyR2 and promoting calcium-activated calcium release (Zhao et al., 2017). This process may be caused by the reduction of PP1 and PP2A levels.

RyR2 phosphorylation caused by CaMKII (Ser-2814) is more critical than that by PKA. Studies have shown that atrial fibrillation induced in FKBP12.6 KO mice can be achieved by inhibiting CaMKII phosphorylation RyR2 instead of PKA, thereby inhibiting SR calcium leakage and delayed depolarization (Bito et al., 2013). Decreasing RyR2 open frequency has been shown to reduce heart failure. The overexpression of cardiac FKBP12.6 in the mouse model has shown to reduce the probability of RyR2 opening, but this method is not sufficient to prevent and mitigate remodeling after myocardial infarction. Besides, TGF-β has a protective effect on MI/R injury, and FKBP12 is a ligand of TGF-β. FKBP12 can also promote the production of interleukin-2, which is used as a transcription factor against MI/R injury (Åström-Olsson et al., 2009). Therefore, FKBP12.6 reportedly has potential in treatment of cardiovascular diseases.

### Calcium Recapture

After myocardial contraction, intracellular Ca^2+^ is returned to SR through SERCA; excessive Ca^2+^ can also be expelled from cells through the NCX, resulting in myocardial cell relaxation (Zhu et al., 2017).

#### NCX

Mammals have three NCX gene types, namely, SLC8A1 (NCX1), SLC8A2 (NCX2), SLC8A3 (NCX3) (Giladi et al., 2016). In mammals, NCX1 is widely distributed in a variety of cells, NCX2 primarily in brain tissue, and NCX3 mainly in brain and skeletal muscle. Besides, SLC8A4 (NCX4) is expressed in amphibians, reptiles, and fish. NCX regulates myocardial ECC, long-term enhancement, neuron development, immune response, and mitochondrial function. NCX structure mainly consists of 10 transmembrane helices and a large cytosolic regulatory loop (f-loop) (Khananshvili, 2013). Unlike SERCA (high affinity-low volume), NCX (low affinity-high volume) responds quickly to changes in intracellular Ca^2+^ based on [Ca^2+^]_i_. NCX performs intracellular and external ion-exchange, according to 3Na^+^:Ca^2+^ (Chu et al., 2016; Giladi et al., 2016). The direction of calcium movement depends on [Na^+^]_i_, [Ca^2+^]_i_, and membrane potential inside and outside the cell. NCX cannot be phosphorylated by kinases but can be reversibly regulated by cellular effectors, including Ca^2+^, Na^+^, H^+^, NO, PIP_2_, phosphoarginine, phosphocreatine, ATP, endogenous NCX inhibitor, and f-loop (Khananshvili, 2013).

NCX participates intracellular calcium homeostasis regulation and is implicated in the occurrence and development of various diseases, such as heart failure, arrhythmia, diabetes, hypertension, and cerebral ischemia. The role of NCX in cardiovascular diseases is debatable. Some studies have shown that heart-specific NCX knockout mice can resist MI/R injury, which may be associated with calcium overload inhibition (Chu et al., 2016). Other studies have shown that NCX can alleviate cardiomyocyte function in patients with heart failure and overexpression reduces the development of systolic and diastolic dysfunction (Khananshvili, 2013). In conclusion, inhibiting reverse transport of NCX is an ideal method for reducing calcium overload and a promising treatment strategy for MI/R.

#### SERCA

There are three SERCA genotypes: ATP2A1 (SERCA1a, SERCA1b), ATP2A2 (SERCA2a, SERCA2b, SERCA2c), and ATP2A3 (SERCA3a-f), each of containing multiple subunits. SERCA2a is the most important, mainly expressed in the heart and slow-twitch skeletal muscle, and mainly involved in cardiomyocyte ECC. SERCA1 is mainly expressed in fast-twitch skeletal muscle, SERCA2b mainly in smooth muscle and non-muscle tissue, SERCA2c mainly in epithelial and hematopoietic cells and SERCA3 primarily in hematopoietic cells (Lipskaia et al., 2014). SERCA has a molecular weight of 110 kDa and hydrolyzes one ATP to transport two Ca^2+^ back to the SR, causing myocardial relaxation (Balcazar et al., 2018). SERCA consists of three distinct domains, including the cytoplasmic head, the transmembrane region of ten helical segments that contain two Ca^2+^ binding sites, and luminal loops (Stammers et al., 2015).

A variety of molecules regulate SERCA; SERCA also affects intracellular calcium homeostasis. Phospholamban (PLB) phosphorylation can release the inhibitory effect on SERCA; PKA affects SERCA sensitivity to calcium (Hayward et al., 2015). SERCA overexpression increases calcium reuptake and calcium transients, but has no effect on calcium leakage caused by RyR. Hence, SERCA agonists are used to treat heart failure, diabetes, and metabolic diseases (Bassani and Bassani, 2005). Studies have shown that soluble guanylyl cyclase activation can stimulate cGMP signals, increase Ca^2+^ uptake by increasing SERCA activity, and reduce reperfusion-induced calcium overload. SERCA is an essential calcium homeostasis and is associated with the pathological mechanisms of various diseases; SERCA is expected to become an important target for cardiovascular disease.

#### PLB

PLB located in SR contains 52 amino acids (6.1 kD), easily forms a polymer (22 kD), which interacts with SERCA and inhibits its activity (Ablorh and Thomas, 2015). PLB consists of three parts: cytoplasmic domain a (including phosphorylation sites serine16 and threonine17), cytoplasmic domain b (rich in amidated amino acids), and domain c (transmembrane structure). PLB can be phosphorylated by PKA (serine16) and CaMKII (threonine17), thereby releasing its inhibitory effect on SERCA (Haghighi et al., 2014). When cytosolic calcium levels are low, PLB combines with SERCA2a to reduce calcium reuptake; when cytosolic calcium levels iare high, CaMKII or PKA can phosphorylate PLB, thereby promoting PLB and SERCA2a depolymerization and allowing SERCA2a to uptake calcium again (Frank and Kranias, 2000). Sarcolipin is a functional PLB homolog with a serine/threonine kinase 16 phosphorylation site (Bhupathy et al., 2007). SERCA activity is super-inhibited by the synergistic action of PLB and SLN (Morita et al., 2008). Disrupting PLB–SERCA2a interactions can reduce cardiac systolic dysfunction, but eliminating PLB is not useful in treating all forms of heart failure (MacLennan and Kranias, 2003). Further research on the structure and function of PLB–SERCA2a will provide new insights into the role of PLB in cardiovascular disease.

#### CaMKII

CaMKII has four genotypes α, β, γ, and δ; α and β are mainly expressed in the nervous system, and γ and δ generally in various cells (Grueter et al., 2006). Among them, δ is the most prevalent subtype in cardiac tissue. CaMKII structure includes amino-terminal catalytic, the central regulatory, and the carboxy-terminal associated domains that oligomerize (Grueter et al., 2006). CaMKII holoenzyme is a wheel-like structure composed of 6-12 homologous or heterologous kinase subunit. CaMKII can act on Ca^2+^-releasing proteins such as RyR2, LTCC, IP3R, and regulate calcium recalculation by PLB and SERCA phosphorylation (Maier and Bers, 2002). CaMKII phosphorylation promotes maintenance of high active LTCC, resulting in excessive extracellular influx into myocardial cells (Mattiazzi and Kranias, 2014). The effect of CaMKII on RyR2 is controversial, as RyR2-mediated increased and decreased calcium release has been reported. CaMKII phosphorylates PLB, which in turn releases its inhibitory effect on SERCA and enhances calcium reuptake capacity.

A study of an animal model of structural heart disease proved that CaMKII overexpression can lead to myocardial dilatation, dysfunction, and calcium homeostasis imbalance in cardiomyocytes (Grueter et al., 2006). βAR increases CaMKII activity and expression. Therefore, CaMKII inhibition can combat cardiac hypertrophy, cardiac dilatation, and myocardial infarction-induced dysfunction, and βAR overstimulation (Mattiazzi and Kranias, 2014). In MI/R injury, CaMKII could activate NF-κb, promote inflammatory response leading to apoptosis, and aggravate MI/R injury (Ling et al., 2013). In simple terms, CaMKII has a significant impact on calcium cycling and various diseases, and it is worth carrying out an in-depth study.

### Calcium Storage

The main cardiomyocyte Ca^2+^ storing organelles are SR and mitochondria. SR and mitochondria are essential during heart contraction and relaxation and provide enough oxygen and ATP to body tissues *via* blood circulation. Although the functional characteristics of SR and mitochondria are different, their cooperation also ensures normal heart operation. Therefore, both mitochondrial and SR reductions and dysfunction can cause heart-related diseases (Chernorudskiy and Zito, 2017).

#### Sarcoplasmic Reticulum

Myocardial SR is composed of longitudinal tubes and terminal cisternae. The longitudinal tube membrane mainly contains SERCA and PLB for calcium reuptake. The terminal cisternae (RyR2) stores and releases Ca^2+^. There are also several soluble proteins in the SR cavity, including calsequestrin and calreticulin. They have low Ca^2+^ affinity, but a large binding capacity. Their primary function is to bind Ca^2+^ in the SR cavity. These proteins can reduce [Ca^2+^]_i_ in the SR cavity, facilitate transport of calcium pumps, and store Ca^2+^ for release at the subsequent myocardial contraction. Therefore, SR regulates cytosolic [Ca^2+^]_i_ mainly in three steps such as calcium release, reuptake, and storage, and different proteins perform their functions (Bers, 2008).

Studies have found that SR calcium balance is very vital for normal physiological function and information transmission of cardiomyocytes. SR calcium homeostasis is closely related to the development of cardiovascular diseases, such as ischemic heart disease, cardiac hypertrophy, hypertension, and heart failure (Fiolet and Baartscheer, 2000). Ca^2+^ release other than that in the normal calcium cycle is called SR calcium leakage, which is mainly mediated by RyR2. Calcium leakage can cause cardiac systolic dysfunction by reducing effective SR Ca^2+^ release, cause diastolic dysfunction by increasing diastolic cytosolic Ca^2+^, and induce arrhythmias (Hulot et al., 2012). Therefore, further study on SR calcium regulatory channels will help reveal the role of Ca^2+^ regulation in cardiovascular disease and provide new insights and strategies for cardiovascular disease treatment and prognosis.

#### Mitochondria

A double membrane structure characterizes the mitochondria; there is a cavity between both membranes due to the folding of inner membrane to form ridges. Mitochondria are the main sites for intracellular oxidative phosphorylation and adenosine triphosphate (ATP) formation. Additionally, mitochondria are also involved in processes such as apoptosis, signal transduction, cell proliferation, and metabolism (Williams et al., 2015). The cardiomyocyte activity is energy consuming. The mitochondria of each ventricular muscle cell account for about 33% of cell volume, which also reflects their importance for cardiomyocytes. Ca^2+^ enters the mitochondrial matrix through a highly selective and low-conductivity mitochondrial calcium uniporter channel. It is pumped into the membrane space through the mitochondrial sodium-calcium exchanger, which maintains calcium dynamic balance in the mitochondria (Williams et al., 2015). Calcium directly or indirectly regulates many components in ATP production, such as crucial enzymes and proteins of the tricarboxylic acid cycle.

Mitochondria are not involved in myocardial ECC regulation under physiological conditions. However, due to the large calcium capacity of mitochondria, which accounts for about 30% of the calcium capacity in plasma and tissue cells, they compensate by alleviating intracellular calcium accumulation during calcium overload in myocardial cells (Dorn and Maack, 2013). Studies have shown that mitochondrial reactive oxygen species production is an early trigger for MI/R injury and causes respiratory chain dysfunction. In I/R heart, mitochondrial channel dysfunction of myocardial cells causes changes in mitochondria membrane potentials (Chouchani et al., 2014). This extends to the entire cell, causing structural and functional disorders. Therefore, mitochondrial damage causes ATP depletion, which accelerates myocardial cell death and promotes the reversible damage of myocardial tissue to irreversible damage (Paradies et al., 2018)

## Interaction of Calcium Overload and Myocardial Ischemia-Reperfusion

### MI/R Protection Signaling Pathway

Ca^2+^ is driven by a large electrochemical gradient across the plasma membrane into the cells. Cells use this external source of signal Ca^2+^ by activating various entry channels, such as voltage-operated channels (Shaw and Colecraft, 2013). The other principal source of Ca^2+^ for signaling is the internal stores that are located primarily in the SR, and IP3R, RyR, and SERCA regulate the release and recycle of Ca^2+^ (Chernorudskiy and Zito, 2017). The principal activator of these channels is Ca^2+^ itself, and this process of Ca^2+^-induced Ca^2+^ release is central to the mechanism of calcium signaling (Bers, 2008). The signaling pathway of MI/R protection is very complicated and involves many factors (Fiolet and Baartscheer, 2000). Obviously, the intervention of the above proteins is potential to promote MI/R protection. And there are some other signaling pathways, which start with G protein-coupled receptors or cytokine receptors activated by adenosine or opioids (Murphy and Steenbergen, 2008). These processes enable protection signals into cardiac cell. Intracellular signals then activate the PI3K-AKT, JAK-STAT, and cGMP-protein kinase G (PKG) pathways. Signaling further activates downstream endothelial nitric oxide synthase (eNOS), glycogen synthase kinase (GSK)-3β, hexokinase II (HK II), and protein kinase Cϵ (PKCϵ). The above process further opens the mitochondrial ATP-dependent potassium channel and inhibits opening of the mitochondrial permeability transition pore (MPTP), which prevents apoptosis factor cytochrome C and apoptosis-inducing factor (AIF) from entering the cytoplasm to induce apoptosis (Hausenloy and Yellon, 2016). In fact, intervention in one of the above pathways may achieve cardiac protection. For example, meprobamate and KAI-9803 (**Table 1**) are two drugs in clinical research, which protect MI/R injury *via* PKC-ϵ activation and PKC-δ inhibition, respectively (Mochly-Rosen et al., 2012). There are insufficient drugs for treating MI/R injury, so it is necessary to conduct further research on MI/R signaling pathways to find targets with cardioprotective effects.

**Table 1:** Summary of MI/R injury drugs.

	Target	Target type	Drugs	KEGG pathway
1	Angiopoietin 1 receptor (TEK)	Clinical trial target	AKB-9778 (Goel et al., 2013)	Ras signaling pathway; Rap1 signaling pathway; HIF-1 signaling pathway; PI3K-Akt signaling pathway; rheumatoid arthritis
2	Adenosine A2b receptor (ADORA2B)	Successful target	Adenosine (Xin et al., 2020)	Calcium signaling pathway; Rap1 signaling pathway; neuroactive ligand-receptor interaction; vascular smooth muscle contraction; alcoholism
3	Adrenergic receptor beta-1 (ADRB1)	Successful target	Metoprolol (Qin et al., 2020)	Calcium signaling pathway; cGMP-PKG signaling pathway; cAMP signaling pathway; neuroactive ligand-receptor interaction; endocytosis; adrenergic signaling in cardiomyocytes; Gap junction; salivary secretion; dilated cardiomyopathy
4	Dipeptidyl peptidase 4 (DPP-4)	Clinical trial target	REC-01(Jackson, 2017)	Protein digestion and absorption; IL2 signaling pathway; TGF-beta receptor signaling pathway
5	Glycoprotein IIb/IIIa receptor (GPIIb/IIIa)	Successful target	Abciximab (Eitel et al., 2013)	Platelet degranulation; elastic fiber formation; PECAM1 interactions; molecules associated with elastic fibers; Integrin cell surface interactions; syndecan interactions; ECM proteoglycans; integrin alphaIIb beta3 signaling; p130Cas linkage to MAPK signaling for integrins; VEGFA-VEGFR2 pathway; MAP2K and MAPK activation
6	Lecithin-cholesterol acyltransferase (LCAT)	Clinical trial target	MEDI6012 (Althaf et al., 2015)	Glycerophospholipid metabolism
7	Leukotriene A-4 hydrolase (LTA4H)	Successful target	Bestatin (W et al., 2018)	Arachidonic acid metabolism; metabolic pathways
8	P2Y purinoceptor 12 (P2RY12)	Successful target	Prasugrel; Brilinta(B, 2010)	Platelet activation
9	Poly [ADP-ribose] polymerase 1 (PARP1)	Successful target	Nicotinamide (EJ et al., 2009)	Base excision repair; NF-kappa B signaling pathway
10	Protein kinase C delta (PRKCD)	Clinical trial target	KAI-9803 (Gross and Gross, 2007)	Chemokine signaling pathway; vascular smooth muscle contraction; tight junction; Fc epsilon RI signaling pathway; Fc gamma R-mediated phagocytosis; neurotrophin signaling pathway; inflammatory mediator regulation of TRP channels; GnRH signaling pathway; estrogen signaling pathway; Type II diabetes mellitus
11	Protein kinase C epsilon (PRKCE)	Clinical trial target	Meprobamate(Mochly-Rosen et al., 2012)	cGMP-PKG signaling pathway; sphingolipid signaling pathway; vascular smooth muscle contraction; tight junction; Fc epsilon RI signaling pathway; Fc gamma R-mediated phagocytosis; inflammatory mediator regulation of TRP channels; type II diabetes mellitus; microRNAs in cancer
12	Proteinase activated receptor 1 (F2R)	Successful target	Vorapaxar (Maki et al., 2010)	Calcium signaling pathway; Rap1 signaling pathway; cAMP signaling pathway; neuroactive ligand-receptor interaction; endocytosis; PI3K-Akt signaling pathway; complement and coagulation cascades; platelet activation; regulation of actin cytoskeleton; pathways in cancer
13	Sodium/hydrogen exchanger (SLC)	Literature-reported target	FR-183998 (Ishizaki et al., 2008)	cAMP signaling pathway; cardiac muscle contraction; adrenergic signaling in cardiomyocytes; regulation of actin cytoskeleton; thyroid hormone signaling pathway; salivary secretion; gastric acid secretion; pancreatic secretion; bile secretion; proteoglycans in cancer
14	Sodium/hydrogen exchanger 1 (SLC9A1)	Clinical trial target	Zoniporide hydrochloride (Tracey et al., 2003)	Calcium signaling pathway; cAMP signaling pathway; cardiac muscle contraction; adrenergic signaling in cardiomyocytes; regulation of actin cytoskeleton; thyroid hormone signaling pathway; salivary secretion; gastric acid secretion; pancreatic secretion; bile secretion; proteoglycans in cancer
15	Thromboxane A2 receptor (TBXA2R)	Successful target	Ridogrel (Xavier et al., 2009)	Calcium signaling pathway; neuroactive ligand-receptor interaction; platelet activation
16	Voltage-gated calcium channel alpha Cav2.1 (CACNA1A)	Successful target	Flunarizine (Wulff et al., 2019)	Calcium signaling pathway; MAPK signaling pathway; synaptic vesicle cycle; retrograde endocannabinoid signaling; glutamatergic synapse; long-term depression; type II diabetes mellitus; morphine addiction; nicotine addiction

### Mechanism of Calcium Overload Generation During MI/R

After a period of ischemia and hypoxia, myocardial cells have metabolic abnormalities, including increased anaerobic fermentation. Furthermore, intracellular H^+^ aggregation causes a low intracellular pH, and intracellular Na^+^ increases through H^+^/Na^+^ exchange (HNX). Excessive intracellular Na^+^ will promote Na^+^ excretion and Ca^2+^ intake by NCX, which significantly increases intracellular calcium levels, hence, leading to calcium overload. When blood flow and oxygen supply to the cardiac tissue returns to normal, extracellular pH level is further increased, HNX and NCX activities are enhanced, and intracellular calcium overload is further aggravated (Murphy and Steenbergen, 2008). Direct H^+^/Ca^2+^ exchange can also directly cause calcium overload. Elevated intracellular calcium levels also activate intracellular calcium-activated calcium release. Existing studies have shown that NCX and HNX inhibition has a protective effect on MI/R injury (Mozaffari et al., 2013). Two candidate drugs, FR-183998 and zoniporide hydrochloride which target HNX, are currently under development for MI/R injury treatment(Tracey et al., 2003; Ishizaki et al., 2008). Opening RyR2 channel effluxes SR Ca^2+^ into the cytoplasm, exacerbating calcium overload. Calcium overload can cause a series of irreversible cell injury responses, such as cardiac contractile dysfunction and apoptosis.

Additionally, MI/R produces some toxic substances, such as oxygen-free radicals (OFR), in the myocardial cells. OFR can decompose cell membrane phospholipid components and damage membrane structure, which leads to increased membrane permeability and excessive extracellular Ca^2+^ influx (Fiolet and Baartscheer, 2000). OFR also damages to the SR membrane, eventually increasing intracellular calcium levels and further exacerbating calcium overload (Mozaffari et al., 2013).

### Mechanism of Calcium Overload Aggravating MI/R Injury

Ca^2+^ plays a vital role in cardiac ECC, so calcium overload will further exacerbate the degree of MI/R injury in cardiac muscle cells. Excessive intracellular calcium will enter the mitochondria, resulting in mitochondrial calcium overload; this inhibits ATP production, exacerbates energy metabolism disorders, and eventually leads to myocardial cell apoptosis (Dorn and Maack, 2013). Mitochondrial damage is regarded as a sign of myocardial cell transformation from reversible to irreversible damage (Williams et al., 2015).

Intracellular calcium can also activate some phospholipases, mainly protein kinase C and phospholipase A, which destroy the cell membrane skeleton. Furthermore, the reaction produces some toxic substances such as free fatty acids, leukotrienes, prostaglandins, and oxygen-free radicals (Hausenloy and Yellon, 2016). These cause mitochondrial dysfunction, increase membrane permeability, impede signal transmission in cells, and promote vast myocardial cell apoptosis. Excessive calcium can also activate caspase, calpain, endonuclease, and phospholipase, which induce intracellular digestion of proteins and fats (Chouchani et al., 2014; Lesnefsky et al., 2017).

Myocardial calcium overload can also cause changes in the structure and function of coronary blood vessels and microvascular endothelial cells. It causes adhesion, accumulation, and infiltration of neutrophils; a series of inflammatory factors release; and further the heart vascular tissue damage (Kleinbongard et al., 2012). Energy metabolism disorder caused by calcium overload can also cause myocardial spasm, and cause pathological changes, such as arrhythmia (Zile and Gaasch, 2011). Therefore, calcium overload plays a vital role in MI/R injury occurrence and development and will also play a key role in preventing and treating MI/R injury.

## Comprehensive Analysis of MI/R Injury and Calcium Signaling Pathway

### MI/R Injury Drugs

Tissue ischemia is due to insufficient hemoperfusion under various pathological conditions. Moreover, MI/R injury is characterized by increased tissue and cell dysfunction, metabolic damage, and structural damage after blood supply is restored and reperfusion occurs (Murphy and Steenbergen, 2008; Lesnefsky et al., 2017). Studies have shown that MI/R causes cardiac dysfunction as well as peripheral vascular injury. Recently, research on the mechanism of MI/R injury and the search for effective measures to reduce reperfusion injury have become hot spots in the cardiovascular field. To more fully understand and summarize research findings and clinical use of MI/R drugs, we obtained relevant information (including target, disease, drug, and KEGG pathway) *via* “Therapeutic Target Database” and verified it with “PubMed” database. Kyoto Encyclopedia of Genes and Genomes (KEGG) pathway is a database that integrates genomic, chemical, and system function information, linking gene catalogs from genomes that have been fully sequenced to higher-level system functions at cell, species, and ecosystem levels (Kanehisa et al., 2017).

In **Table 1**, we have summarized drugs and candidates for MI/R injury treatment. Over half of the drugs are marketed, three eighth are drug under investigation, and the remaining are potential candidates. Nearly half of the drugs target calcium regulators that are mainly involved in calcium signaling pathway regulation. Except for calcium channels, MI/R targets include angiopoietin 1 receptor, P2Y purinoceptor 12, protein kinase C, lecithin-cholesterol acyltransferase, and dipeptidyl peptidase 4, indicating complexity of MI/R injury mechanisms and the diversity of targets. In fact, the drugs used to treat MI/R injury are very limited in clinical practice, and most of the drugs are selected according to the symptoms (Ibanez et al., 2015). Some drugs, such as prasugrel, brilinta, and AKB-9778, prevent blood vessels from being clogged due to platelet activation or vasodilation (B, 2010; Goel et al., 2013), and some drugs, such as flunarizine, meprobamate, and KAI-9803, reduce cardiomyocyte damage by calcium overload and PKC pathway inhibition (Mochly-Rosen et al., 2012). Furthermore, according to the KEGG pathway, most drugs are not involved in a single signal pathway, but in an interconnected signal network, which also reflects the intricate connection between various reactions in the body. This also helps us to understand the role of drugs in signaling networks and links between disease and various signaling pathways. Some of the drugs in **Table 1** are still at different stages of research, so basic and clinical researchers need to work rigorously to develop more safe and effective drugs to alleviate the pain and distress of MIR patients.

### Calcium Signaling Pathway Drugs

We also searched for the calcium signaling pathway-related drugs using the “Therapeutic Target Database” to better understand the role of calcium homeostasis in various diseases. As shown in **Table 2**, calcium signaling pathway drugs are mainly used to treat cardiovascular and cerebrovascular diseases, such as myocardial (cerebral) I/R injury, hypertension, cardiac failure, and myocardial infarction. About half of the drugs are marketed, about two fifths are undergoing clinical trials, and the remaining are potential active candidates.

**Table 2:** Summary of calcium signaling pathway drugs.

	Target	Target type	Disease	Drugs	KEGG pathway
1	Adenosine A1 receptor (ADORA1)	Clinical trial target	Orthostatic hypotension	Caffeine (Rosenthal et al., 2008); Tonapofylline (Chen and Tang, 2007)	Calcium signaling pathway; Rap1 signaling pathway; cAMP signaling pathway; neuroactive ligand-receptor interaction; vascular smooth muscle contraction; Parkinson's disease; alcoholism
2	Adenosine A2a receptor (ADORA2A)	Clinical trial target	Coronary artery disease	Apadenoson; Binodenoson(Hodgson et al., 2007)	Calcium signaling pathway; Rap1 signaling pathway; cAMP signaling pathway; neuroactive ligand-receptor interaction; vascular smooth muscle contraction; Parkinson's disease; alcoholism
3	Adenosine A2b receptor (ADORA2B)	Clinical trial target	Hypertension	YT-146 (T et al., 1994)	Calcium signaling pathway; Rap1 signaling pathway; neuroactive ligand-receptor interaction; vascular smooth muscle contraction; alcoholism
4	Adenosine A2b receptor (ADORA2B)	Successful target	Paroxysmal supraventricular tachycardi; reperfusion injury	Adenosine (Xin et al., 2020)	Calcium signaling pathway; Rap1 signaling pathway; neuroactive ligand-receptor interaction; vascular smooth muscle contraction; alcoholism
5	Adrenergic receptor beta-1 (ADRB1)	Successful target	Hypertension; reperfusion injury	Metoprolol (Qin et al., 2020)	Calcium signaling pathway; cGMP-PKG signaling pathway; cAMP signaling pathway; neuroactive ligand-receptor interaction; endocytosis; adrenergic signaling in cardiomyocytes; gap junction; salivary secretion; dilated cardiomyopathy
6	Angiotensin II receptor type-1 (AGTR1)	Successful target	Hypertension	Valsartan (Chang et al., 2020)	Calcium signaling pathway; cGMP-PKG signaling pathway; neuroactive ligand-receptor interaction; adrenergic signaling in cardiomyocytes; vascular smooth muscle contraction; renin-angiotensin system; renin secretion; pathways in cancer
7	Calcium channel unspecific (CaC)	Successful target	Cerebral vasospasm; hyperinsulinemia	Nimodipine (G and M, 2009)	Calcium signaling pathway; MAPK signaling pathway; cardiac muscle contraction;
8	Calcium-activated potassium channel KCa1.1 (KCNMA1)	Literature-reported target	Asthma	Cromoglycate lisetil hydrochloride (Law et al., 2011)	Calcium signaling pathway; cGMP-PKG signaling pathway; vascular smooth muscle contraction; Insulin secretion; salivary secretion; pancreatic secretion
9	Calcium-release activated calcium channel (CRACM)	Clinical trial target	Non-Hodgkin lymphoma	RP4010 (Cui et al., 2018)	Calcium signaling pathway; cAMP signaling pathway; platelet activation; primary immunodeficiency
10	Calpain-2 (CAPN2)	Clinical trial target	Cerebral infarction	ABT-957 (Ge et al., 2005)	Protein processing in endoplasmic reticulum; apoptosis; calcium signaling pathway; Alzheimer's disease
11	CaM-kinase II (CAMK2)	Clinical trial target	Anxiety disorder	Rimacalib (J et al., 2009)	Calcium signaling pathway; ErbB signaling pathway; cAMP signaling pathway; HIF-1 signaling pathway; Wnt signaling pathway; circadian entrainment; neurotrophin signaling pathway; cholinergic synapse; Inflammatory mediator regulation of TRP channels; Insulin secretion; GnRH signaling pathway; melanogenesis; oxytocin signaling pathway; glucagon signaling pathway; gastric acid secretion
12	Endothelin A receptor (EDNRA)	Successful target	Cerebrovascular disease; hypotension	Ambrisentan (Rosenzweig, 2006)	Calcium signaling pathway; cGMP-PKG signaling pathway; cAMP signaling pathway; neuroactive ligand-receptor interaction; vascular smooth muscle contraction; renin secretion; pathways in cancer
13	Nitric-oxide synthase endothelial (NOS3)	Clinical trial target	Pulmonary hypertension; brain injury	Tilarginine acetate (Investigators et al., 2007)	Calcium signaling pathway; arginine and proline metabolism; Metabolic pathways; cGMP-PKG signaling pathway; HIF-1 signaling pathway; sphingolipid signaling pathway; PI3K-Akt signaling pathway; VEGF signaling pathway; platelet activation; estrogen signaling pathway; oxytocin signaling pathway
14	Proteinase activated receptor 1 (F2R)	Successful target	Myocardial infarction	Vorapaxar (Maki et al., 2010)	Calcium signaling pathway; Rap1 signaling pathway; cAMP signaling pathway; neuroactive ligand-receptor interaction; endocytosis; PI3K-Akt signaling pathway; complement and coagulation cascades; platelet activation; regulation of actin cytoskeleton; pathways in cancer
15	S100 calcium-binding protein B (S100B)	Clinical trial target	Stroke; type 2 diabetes;	ONO-2506 (Asano et al., 2005)	Calcium signaling pathway; cGMP-PKG signaling pathway; neuroactive ligand-receptor interaction endocytosis; salivary secretion
16	Sarcoplasmic/endoplasmic reticulum calcium ATPase (ATP2A)	Successful target	Malaria	Artemisinin (Choia et al., 2008)	Calcium signaling pathway; pyrimidine metabolism; metabolic pathways
17	Sarcoplasmic/endoplasmic reticulum calcium ATPase 2 (ATP2A2)	Clinical trial target	Hypertension; asthma	Gallopamil (Sulpizio et al., 2005)	Calcium signaling pathway; renin-angiotensin system; Alzheimer's disease; adrenergic signaling in cardiomyocytes; Thyroid hormone signaling pathway; Alzheimer's disease; hypertrophic cardiomyopathy (HCM); arrhythmogenic right ventricular cardiomyopathy (ARVC); dilated cardiomyopathy
18	Sodium/calcium exchanger (SLC)	Clinical trial target	Cardiovascular disease	CALDARET HYDRATE(Kawasumi et al., 2007)	Calcium signaling pathway; cardiac muscle contraction; adrenergic signaling in cardiomyocytes;
19	Sodium/hydrogen exchanger 1 (SLC9A1)	Clinical trial target	Myocardial infarction; angina pectoris	Zoniporide hydrochloride(Tracey et al., 2003)	Calcium signaling pathway; cAMP signaling pathway; cardiac muscle contraction; adrenergic signaling in cardiomyocytes; regulation of actin cytoskeleton; thyroid hormone signaling pathway; salivary secretion; gastric acid secretion; pancreatic secretion; bile secretion; proteoglycans in cancer
20	Thromboxane A2 receptor (TBXA2R)	Successful target	Acute myocardial infarction	Ridogrel(Xavier,Davel,Fukuda and Rossoni 2009)	Calcium signaling pathway; neuroactive ligand-receptor interaction; platelet activation
21	Voltage-gated calcium channel alpha Cav1.2 (CACNA1C)	Successful target	Hypertension	Rauwolfia serpentina root(Wiens and De Luca, 2016)	Calcium signaling pathway; MAPK signaling pathway; cGMP-PKG signaling pathway; cAMP signaling pathway; cardiac muscle contraction; adrenergic signaling in cardiomyocytes; vascular smooth muscle contraction; circadian entrainment; type II diabetes mellitus; hypertrophic cardiomyopathy (HCM); Arrhythmogenic right ventricular cardiomyopathy (ARVC); dilated cardiomyopathy
22	Voltage-gated calcium channel alpha Cav1.2 (CACNA1C)	Clinical trial target	Alzheimer disease	ARC029 (H et al., 1989)	Calcium signaling pathway; MAPK signaling pathway; cGMP-PKG signaling pathway; cAMP signaling pathway; cardiac muscle contraction; adrenergic signaling in cardiomyocytes; vascular smooth muscle contraction; circadian entrainment; Insulin secretion; type II diabetes mellitus; hypertrophic cardiomyopathy (HCM); arrhythmogenic right ventricular cardiomyopathy (ARVC); dilated cardiomyopathy
23	Voltage-gated calcium channel alpha Cav2.1 (CACNA1A)	Successful target	Cardiac failure; reperfusion injury	Flunarizine (Wulff et al., 2019)	Calcium signaling pathway; MAPK signaling pathway; synaptic vesicle cycle; retrograde endocannabinoid signaling; glutamatergic synapse; long-term depression; type II diabetes mellitus; morphine addiction; nicotine addiction
24	Voltage-gated calcium channel alpha Cav2.2 (CACNA1B)	Successful target	Pain; traumatic brain injury	Ziconotide (Xiong et al., 2009)	Calcium signaling pathway; MAPK signaling pathway; synaptic vesicle cycle; retrograde endocannabinoid signaling; GABAergic synapse; dopaminergic synapse; taste transduction; type II diabetes mellitus
25	Voltage-gated calcium channel alpha Cav3.1 (CACNA1G)	Successful target	Hypertension; Insomnia;	Verapamil (TD et al., 2005)	Calcium signaling pathway; MAPK signaling pathway; circadian entrainment; type II diabetes mellitus
26	Voltage-gated calcium channel alpha Cav3.2 (CACNA1H)	Successful target	Metabolic acidosis; migraine	Sodium bicarbonate (Wood et al., 2009)	Calcium signaling pathway; MAPK signaling pathway; circadian entrainment
27	Voltage-gated calcium channel alpha-2/delta-1 (CACNA2D1)	Successful target	Hypertension	Diltiazem (Huang et al., 2009); Amlodipine (Ram, 2009)	Calcium signaling pathway; MAPK signaling pathway; cardiac muscle contraction; adrenergic signaling in cardiomyocytes; Oxytocin signaling pathway; hypertrophic cardiomyopathy (HCM); arrhythmogenic right ventricular cardiomyopathy (ARVC); dilated cardiomyopathy
28	Voltage-gated calcium channel alpha-2/delta-1 (CACNA2D1)	Successful target	Chronic obstructive pulmonary disease	Pregabalin (Andrade, 2018)	Calcium signaling pathway; MAPK signaling pathway; cardiac muscle contraction; Adrenergic signaling in cardiomyocytes; Oxytocin signaling pathway; hypertrophic cardiomyopathy (HCM); arrhythmogenic right ventricular cardiomyopathy (ARVC); dilated cardiomyopathy
29	Voltage-gated L-type calcium channel (L-CaC)	Successful target	Angina pectoris; hypertension	Nifedipine (Kleinsasser and Loeckinger, 2002); Nisoldipine; Levamlodipine(Oh et al., 2012)	Calcium signaling pathway; MAPK signaling pathway; Cardiac muscle contraction;
30	Voltage-gated L-type calcium channel (L-CaC)	Clinical trial target	Cardiovascular disease	BAY-Y-5959 (GH et al., 1998)	Calcium signaling pathway; Cardiac muscle contraction;

Calcium channel blockers (CCBs), such as nifedipine, nisoldipine, diltiazem, verapamil, nimodipine, and amlodipine (**Table 2**), can be widely used to treat cardiovascular diseases including myocardial infarction, hypertension, arrhythmia, and angina pectoris. CCBs reduce calcium levels in myocardial cells, which triggers a series of physiological effects against multiple diseases. Most CCBs have significant effects and reasonable prices, but there are still many adverse reactions. CCBs only target angina pectoris, caused by coronary spasm, but are not effective against other types of angina pectoris (Capiod, 2011; Kleinbongard et al., 2012). Therefore, for, appropriate drugs should be selected for treating different patients, according to the disease type, to reduce adverse reactions as much as possible. In addition, calcium overload aggravates cerebral I/R injury, and drugs targeting calcium channels and calcium pathway-related factors have good clinical effects on cerebral I/R injury (Ge et al., 2005; Zhao et al., 2013). The drug targets are mainly voltage-dependent calcium channels, calcium-activated ion channels, and adenosine receptors. According to KEGG analysis, the drugs listed in **Table 2** also involve multiple signaling pathways. In addition to the calcium pathway, other pathways, such as the cAMP signaling pathway, Adrenergic signaling pathway, cardiac muscle contraction, platelet activation, also occur more frequently, and there is an upstream and downstream connection between these pathways and the calcium signaling pathways. Cardiac muscle contraction is a complex process initiated by the electrical excitation of cardiac myocytes, which is the processes of ECC (Bers, 2008). In other signaling pathways, calcium serves as an important second messenger. Adrenergic receptor as the pre-dominate receptor, induces positive inotropic and chronotropic effects, the most effective mechanism to acutely increase output of the heart, by coupling to Gs, formation of cAMP by adenylyl cyclase, and PKA-dependent phosphorylation of various target proteins (such as RyR2, PLB, and LTCC) (Qin et al., 2020). During platelet activation, different receptors are stimulated by various agonists, almost converging in increasing intracellular Ca^2+^ concentration that stimulate platelet shape change and granule secretion (Maki et al., 2010). In a word, some important proteins, such as RyR2, PLB, FKBP12.6, and NCX, participate in the calcium cycling process and maintain calcium homeostasis, thereby ensuring that intracellular Ca^2+^ transfers different signals to downstream signaling pathways. Therefore, the importance of calcium pathways is self-evident, and future research should focus on calcium pathway drugs for treating cardiovascular and cerebrovascular diseases.

### Comprehensive Analysis

As a second messenger in cells, Ca^2+^ plays an essential role in maintaining cell proliferation, division, and energy metabolism. The calcium signal pathway-related protein expression and ion channel regulation mechanism connect arrhythmia, myocardial hypertrophy, and heart failure in cardiovascular diseases, making the development of these diseases a cause and effect (Bers, 2008; Hidalgo, 2017). As MI/R injury-mediated calcium overload is multifactorial, it is vital to regulate intracellular calcium homeostasis to maintain normal physiological function and information transmission in cells (Fiolet and Baartscheer, 2000). Based on data of tables, about two fifths of diseases (**Table 1**) involve calcium signaling pathways, three fifth of the drugs (**Table 2**) are used to treat cardiovascular disease, and more than one-fifth of the drugs (**Table 2**) to treat MI/R injury. The above results also prove that calcium homeostasis plays an irreplaceable role in normal cell operation and cardiovascular disease development, especially MI/R injury and hypertension.

**Figure 1:**
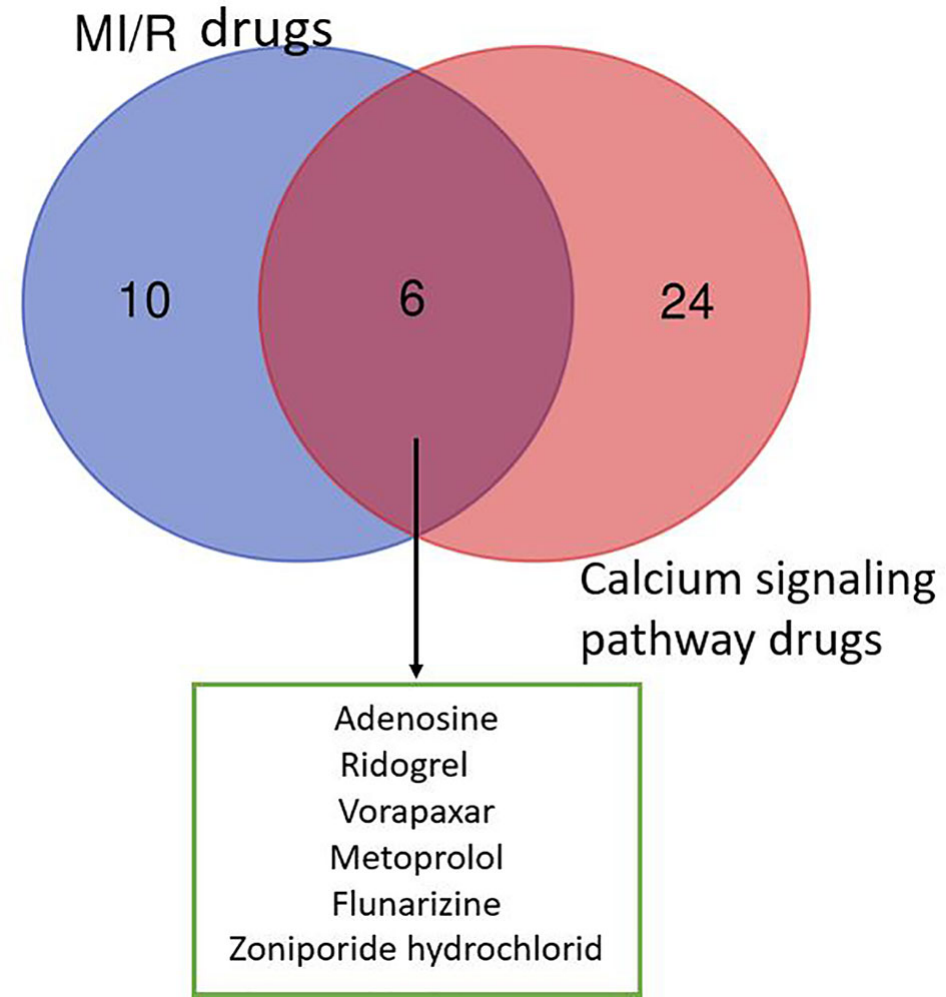
Venn diagram of drugs from MI/R injury and calcium signaling pathway. We collected 16 drugs for the treatment of MIR injury and 30 drugs involved in calcium signaling pathway. The MI/R drugs related to calcium signaling pathway include: adenosine, ridogrel, vorapaxar, metoprolol, flunarizine, and zoniporide hydrochloride.

As shown in **Figure 1**, the six drugs including adenosine, ridogrel, vorapaxar, metoprolol, flunarizine, and zoniporide hydrochloride have the potential to treat MIR, but their treatment mechanisms are different. Our analysis of the data in **Tables 1** and **2** shows that voltage-dependent calcium channels, adrenergic receptor β1, proteinase-activated receptor 1 (PAR1), HNX, adenosine A2b receptor, and thromboxane A2 (TXA2) receptor are important targets for MI/R injury treatment and calcium pathways. As mentioned in *Mechanism of Calcium Overload Generation During MI/R*, when MI/R, a huge difference in intracellular and extracellular pH of the myocardial cells occurs, and HNX and NCX change the movement of H^+^, Na^+^, and Ca^2+^, which aggravates calcium overload (Chu et al., 2016). The role of LTCC as ECC activating signal in the calcium pathway is self-evident. Besides, adrenergic receptors can effectively induce downstream signaling pathways, including calcium cycling pathways by cAMP formation and PKA phosphorylation, thereby increasing cardiac output (Qin et al., 2020). Studies also have shown that angiotensin-induced increase in TXA2 has a protective effect on MI/R injury, and this regulatory effect is related to maintaining calcium homeostasis (Dogan et al., 1997). Therefore, these targets directly or indirectly play a role in relieving MI/R injury by regulating calcium signaling pathways.

Besides, the drugs we listed are basically chemical drugs, which generally have specific targets. But, the composition of traditional Chinese medicine (TCM) is complex, and the targets are not single. For cardiovascular diseases, which involve multiple signaling pathways, TCM has its unique advantages. For example, *Rauwolfia serpentina* root was clinically proven to treat hypertension, fever, liver disease, and other diseases in the past (Wiens and De Luca, 2016; Dong et al., 2018). Besides, current research also proved its good effect on MI/R injury. Therefore, investing TCM for cardiovascular disease treatment should be increased.

In fact, nearly half of the drugs listed in **Tables 1** and **2** are in preclinical or clinical research, such as FR-183998 and zoniporide. In order to ensure the safety and effectiveness of drugs, drug development is a long and costly process. If there is a problem in any part of the research and development process, the drugs will be forced to stop. Therefore, not all of the drugs we listed can be successfully marketed in the end and solve problems for patients. The disease characteristics of MI/R injury determine that more effective targets and even drugs related to calcium pathways can be discovered in the future. These still need persevering research by scientific researchers.

## Conclusions

Normal heart function depends on coordinated Ca^2+^ movement into and out of the plasma membrane and sarcoplasmic reticulum; calcium circulation disorders can cause various heart diseases. Changes in the Ca^2+^ steady-state, including decreased SR Ca^2+^ reuptake, abnormal calcium channels, SR Ca^2+^ leakage, or significantly decreased SERCA2a expression can cause various heart diseases, including ischemic heart disease, reperfusion injury, hypertrophic cardiomyopathy, diastolic cardiomyopathy, and heart failure (Bers, 2008). With continuous improvement of electrophysiology and pharmacology technology, especially combination and application of patch-clamp technology and molecular cloning technology, researcher can gain an in-depth understanding of drugs and the ion channel effects of cardiovascular active ingredients from cell to the molecular levels. These methods are significant in understanding the impact of drugs on ion channel activity and mechanism of action against MI/R injury. By studying MI/R injury mechanism and better understanding the underlying signaling pathways, we will have better opportunities to develop treatments to protect against MI/R injury. We look forward to the development of new therapies to reduce MI/R injury and incorporate them into clinical trials as soon as possible.

**Figure 2:**
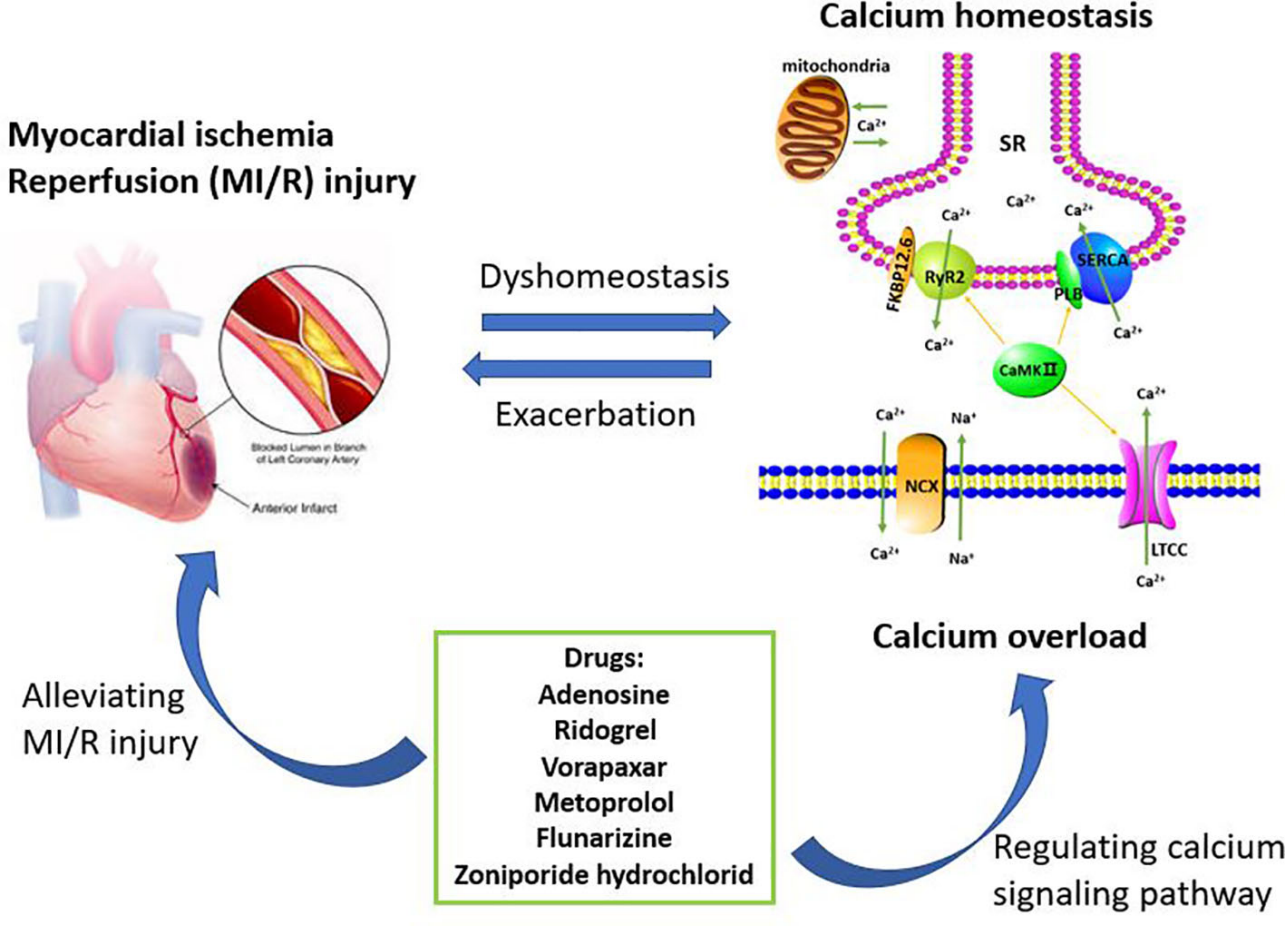
Calcium overload is the important target of treating myocardial ischemia/reperfusion (MI/R) injury. The maintenance of calcium homeostasis requires the participation of multiple regulatory proteins including LTCC, RyR2, SERCA, NCX, PLB, FKBP12.6, and CaMK II. When MI/R occurs, calcium homeostasis will be broken and further developed into calcium overload. Calcium overload further exacerbates MI/R injury. Therefore, inhibiting calcium overload is an effective way to reduce MI/R injury. These drugs currently on the market or under investigation including adenosine, ridogrel, vorapaxar, metoprolol, flunarizine, and zoniporide hydrochloride, are for the treatment of MI/R injury or have the potential to treat MI/R injury. These drugs can play a cardioprotective role by regulating the calcium signaling pathway.

In summary, understanding the role of calcium pathway-related proteins in MI/R injury is of great benefit in elucidating the pathogenesis of MI/R injury. Copious literature summarize that MI/R injury can cause calcium overload, which can further aggravate MI/R injury. Therefore, these regulatory proteins, such as LTCC, NCX, SERCA, RyR2, PLB, and FKBP12.6, provide potential targets for the prevention and treatment of clinical MI/R injury, and multi-target therapy may also have potential application. After analyzing the data in the Tables, we found that six drugs including adenosine, ridogrel, vorapaxar, metoprolol, flunarizine, and zoniporide hydrochloride were used to treat MI/R injury and the treatment mechanisms were related to the calcium signaling pathway (**Figure 2**). The specific targets of these six drugs, such as voltage-dependent calcium channels, adrenergic receptors β1 and HNX are different and involved in calcium signaling pathway, which also reflects the complexity of the MI/R mechanism and the important role of maintaining calcium homeostasis on MI/R injury. In our statistics, some drugs are still in the research stage, and the clinical demand has also pushed researchers to make greater efforts to find more effective and safe drugs to treat MI/R injury. Therefore, this article reviews the role of calcium overload in the development of MI/R injury and the current research progress of marketed and candidate drugs, aiming to provide some help for further studying the protection mechanism and therapeutic reagents of MI/R injury.

However, the current study is still in its initial stage, and there are many problems, mainly with regard to two aspects. Firstly, MI/R injury involves multiple signaling pathways, and the cross-talk between multiple MI/R-related mechanism and calcium regulation can be further studied. Additionally, different calcium pathway drug combinations or multi-target chemical synthetic drugs can be tested in MI/R injury treatment to cope with the complex pathological mechanism underlying MI/R. Second, to study calcium homeostasis in MI/R injury, application and development of new technologies can promote research breakthroughs, such as real-time calcium channel current and calcium concentration detection, and real-time capture detection for calcium channel protein modification, which broaden the scope of basic research and accelerate the search for MI/R candidate drugs.

## Author Contributions

Conceptualization: RW and MW. Writing—original draft preparation: RW. Writing—review and editing: MW, GS, and XS. Visualization: SH. Funding acquisition: GS and XS. All authors contributed to the article and approved the submitted version.

## Funding

This research was funded by the Drug Innovation Major Project (grant number 2018ZX09711001-009), National Key Research and Development Project (grant number 2017YFC1702504 and 2018YFC1707408), and the National Natural Sciences Foundation of China (grant number 81891012).

## Conflict of Interest

The authors declare that the research was conducted in the absence of any commercial or financial relationships that could be construed as a potential conflict of interest.
